# Estimating incidence rates of primary infection and reinfection with hepatitis C virus among people who inject drugs in Scotland: a model-based analysis of repeated cross-sectional survey data

**DOI:** 10.1016/j.lanepe.2025.101505

**Published:** 2025-10-28

**Authors:** Scott A. McDonald, Norah Palmateer, Andrew McAuley, Rory N. Gunson, Stephen T. Barclay, John F. Dillon, Matthew Hickman, Sharon J. Hutchinson

**Affiliations:** a School of Health and Life Sciences, Glasgow Caledonian University, Glasgow, UK; b Public Health Scotland, Edinburgh, UK; c West of Scotland Specialist Virology Centre, NHS Greater Glasgow and Clyde, UK; d Department of Gastroenterology, Glasgow Royal Infirmary, Glasgow, UK; e School of Medicine, University of Dundee, Dundee, UK; f Population Health Sciences, Bristol Medical School University of Bristol, Bristol, UK; g National Drug and Alcohol Research Centre, University of New South Wales, Sydney, Australia

**Keywords:** Hepatitis, Hepatitis C, HCV elimination, Blood borne virus, People who inject drugs, PWID, Injecting drug use, Drug use, Drug misuse

## Abstract

**Background:**

Reducing the incidence of hepatitis C virus (HCV) infection is a World Health Organization (WHO) elimination goal, but approaches to estimate this from population-level survey data are lacking. We modelled HCV incidence among people who inject drugs (PWID) surveyed over time, to provide evidence on whether Scotland has reached the WHO elimination target of ≤2 per 100 person-years in this population.

**Methods:**

A statistical model was fitted using HCV infection data from five sweeps (2013-14, 2015-16, 2017-18, 2019-20, and 2022-23) of a national bio-behavioural survey, involving 11,651 PWID recruited at sites providing injecting equipment. Per-sweep incidence rates of primary chronic HCV infection, reinfection, and combined primary and reinfection (‘total infection’) were inferred within a Bayesian framework. Incidence rates relate to the number of new infections per 100 person-years for the population at risk of (primary, reinfection and total) infection.

**Findings:**

In 2022-23, the model-estimated total, re-infection and primary infection incidence rates were 3.4 per 100 person-years (95% credible interval (CrI):2.6–4.3), 1.9 (1.3–2.6), and 4.1 (3.0–5.4), respectively. For total new infections, the model-estimated incidence rate decreased by 51% from 7.0 per 100 person-years in 2015-16 to 3.4 in 2022-23 (relating to an absolute decrease of 3.6 per 100 person-years; 95% CrI: 2.0–5.3). Between 2015-16 and 2022-23, model-estimated re-infection and primary infection incidence rates decreased by 78% and 40%, respectively.

**Interpretation:**

Over a period when direct-acting antiviral therapy was scaled-up in Scotland, major reductions in the incidence of primary infection, reinfection, and total HCV infection were evident, indicating that the WHO target is within reach, for a relatively high-risk population of PWID.

**Funding:**

Public Health Scotland (for NESI); NIHR HPRU in Behavioural Science and Evaluation; NIHR Programme Grants for Applied Research programme (reference number RP-PG-0616-20008).

**Research in context**

**Evidence before this study**

Approaches are lacking for routinely measuring country-level progress towards the key WHO 2030 target of reducing HCV incidence among people who inject drugs (PWID) to ≤2 infections per 100 person-years. We searched PubMed for publications reporting observational studies, systematic reviews, or meta-analyses from 1 January 2015 through to 18 April 2025, that included the terms "hepatitis C″ AND "incidence" AND ("people who inject drugs” OR “PWID") in any field, without language restrictions. Studies that reported the incidence of either primary HCV infection or total HCV infection (involving both primary infection and reinfection) for the population of PWID in the period since 2015 (the approximate launch of effective direct-acting antiviral (DAA) therapies) were deemed relevant.

Our search of the published literature found two principal approaches to measuring the incidence of primary HCV infection or total infection (aggregating primary infection and reinfection) via: (i) direct estimation (through re-testing of at-risk populations or via the ‘window period’ method based on antibody and RNA test results), and (ii) dynamic or statistical modelling. The most recent data are supplied by a large global study reporting pooled HCV incidence among PWID for the period 2015-2021. This pooled estimate, 8.6 (95% CI: 7.1–10.7) per 100 person-years, was derived by pooling incidence from studies using direct estimation, force-of-infection modelling, and dynamic modelling (for the former two types, estimates were for primary infection only; for the latter, re-infection incidence was assumed to be equal to primary incidence).

**Added value of this study**

This study presents a statistical modelling approach for estimating the incidence of primary infection, reinfection, and total HCV infection that can be applied to routinely available repeated cross-sectional bio-behavioural survey data. Model-based evidence generated for a large population of PWID in Scotland since 2015-16 demonstrated that over a period when DAA therapy was scaled-up in Scotland, major reductions – on the order of 40–78% – in the incidence of HCV infection were evident, with the estimated incidence of primary infection, reinfection, and HCV infection in 2022-23 reaching 4.1, 1.9, and 3.4 per 100 person-years, respectively.

**Implications of all the available evidence**

Major reductions in the population-level incidence HCV infection among PWID associated with the country-level scale-up of DAAs provides compelling evidence of treatment as prevention. To achieve and maintain the WHO HCV incidence target, resources to sustain the provision of effective therapies together with access to harm reduction and testing, to PWID at risk of HCV infection, should be a priority.

## Introduction

Infection with hepatitis C virus (HCV) constitutes a major public health issue, with untreated infection associated with increased risks of liver damage, hepatocellular carcinoma, and premature mortality. There were approximately 50 million people globally living with chronic HCV infection in 2022, with an estimated 240,000 HCV-related deaths and 1.0 million new infections occurring every year.^1^ The recent development of safe and effective direct-acting antiviral (DAA) therapies has been a game-changer in terms of patient management, provided that DAAs are accessible and affordable, and barriers to HCV-infected persons entering the diagnosis, care and treatment pathway can be overcome.^2^ Because of the potential for DAAs to avert the future disease burden of HCV, the WHO has put forward targets to eliminate HCV as a public health threat; namely a reduction between 2015 and 2030 in HCV incidence in people who inject drugs (PWID) to ≤2 per 100 person-years (previously an 80% reduction), and in HCV-related mortality of 65%.^1^^,^^3^

The Scottish Government previously set ambitious targets for the elimination of HCV as a major public health threat by 2025, five years ahead of the WHO target.^4^ The elimination strategy has focused the scale-up of DAA treatment with high-coverage harm reduction (treatment-as-prevention (TasP)) on PWID, the largest transmission risk group in Scotland and other high-income countries.^5^^,^^6^ As it is challenging to directly measure the incidence of new HCV infection, routine monitoring has focused initially on a proxy target for this of a 80% reduction in HCV prevalence among PWID.^7^, ^8^, ^9^ A statistical modelling study using data from the UK demonstrated major reductions (compared to counterfactuals) in HCV prevalence among PWID since 2015,^6^ with the latest findings suggesting that one region of Scotland (Tayside) has met the proxy 80% prevalence reduction target.^8^^,^^10^, ^11^, ^12^

To track progress toward the WHO goal, it is of fundamental importance to monitor not only HCV prevalence but also changes in HCV incidence over time, and to estimate if and when the absolute HCV incidence target of ≤2 new infections per 100 person-years for PWID will be met. As incidence is difficult to measure directly, indirect approaches such as modelling^13^ may offer a way forward, and have been already used by Georgia to support achievement of the WHO absolute HCV incidence target among PWID by 2022.^14^

Besides primary HCV infection incidence, estimation of the incidence of re-infection is important, because with the deployment and scale-up of DAA treatment of actively injecting PWID, the pool of high-risk persons who clear their HCV infection and remain susceptible to reinfection also increases; this could potentially translate to an increasing incidence of re-infection compared with the incidence of primary infection. Scotland is well placed to estimate the incidence of HCV infection using data from the Needle Exchange Surveillance Initiative (NESI), representing a large national population-based serial cross-sectional survey of PWID.^15^^,^^16^ Through statistical modelling of the change in the proportions of NESI respondents with chronic infection across five survey sweeps between 2013-14 and 2022-23 – additionally informed by data on reinfections and reported HCV therapy uptake – our objectives were (i) to estimate primary HCV infection and reinfection incidence rates for the latter four sweeps, (ii) to infer whether incidence rates across successive sweeps were stable, decreasing, or increasing, and (iii) to provide evidence on whether Scotland has reached the WHO chronic HCV elimination target of a reduction in total HCV infection incidence to ≤2 per 100 person-years among PWID since the 2015-16 NESI sweep.

## Methods

We developed a statistical modelling approach, applied to cross-sectional NESI survey and involving integrated HCV testing and treatment data, to infer the per-sweep incidence rates of primary chronic HCV infection and reinfection, and combined primary chronic HCV infection and reinfection (referred to as ‘total infection’ incidence rates). The model essentially mimics the trajectory of a dynamic population of PWID over time, where members of this population can be chronically infected with HCV, cured of their infection, and can become chronically infected again (reinfected). Because transmission events cannot be observed, inference of incidence rates is carried out using all relevant data within a Bayesian framework. Through fitting to the observed proportions of chronically infected respondents, the per-sweep proportions of primary infections, reinfections, and existing chronic infections can be inferred, with accompanying uncertainty.

### Data sources

A national bio-behavioural survey of PWID (NESI) has been conducted on an approximate biennial basis since 2008–09, and with about 100 sites that provide sterile injecting equipment included each sweep, coverage is estimated at 10–15% of Scotland's active PWID population.^15^ NESI data from five sweeps – 2013-14, 2015-16, 2017-18, 2019-20, and 2022-23 – were used, representing a total of 11,651 respondents (relating to 9909 individuals accounting for participation across multiple sweeps). In brief, NESI data collection involves linked elicited questionnaire responses and a dried blood spot, which is tested anonymously for HCV antibody and RNA using laboratory methods detailed elsewhere.^12^ Data for repeat respondents within a given sweep were removed (retaining the first result only; *n* = 142, 124, 74, 66, and 137 responses were removed from each sweep, respectively). Data regarding the proportions of all participants in each sweep stratified by antibody (Ab) test result (positive, negative), RNA test result (positive, negative), and self-reported antiviral therapy status (ever treated, never treated) were used (see Supplementary Materials, Table S1). Following previous research using NESI data, people who tested HCV antibody-negative but with an ‘insufficient’ RNA result (*n* = 212 across all five sweeps) were considered to be Ab-/RNA-.^17^ All other insufficient or indeterminate or missing test results were excluded. To simplify the modelling, we ignore transitions from acute to chronic infection and the very small number of people (*n* = 93 across all five sweeps) with evidence for recent acute infection (i.e., Ab-/RNA+) were also excluded. The total number of participants over the five sweeps is 10,751 (involving a minority of individuals participating in more than one sweep). We compared the characteristics (i.e., sex, age, and recent use of opioid agonist therapy (OAT)) of NESI participants to published estimates of the population of people dependent on opioids in Scotland,^18^ as this represents an inclusive denominator for measuring progress on HCV elimination among PWID.^11^

Ethical approval for NESI was provided by the West of Scotland Research Ethics Committee (reference 08/S0709/46); written informed consent was obtained from each individual participating in the NESI survey.

### Model

**Fig. 1:**
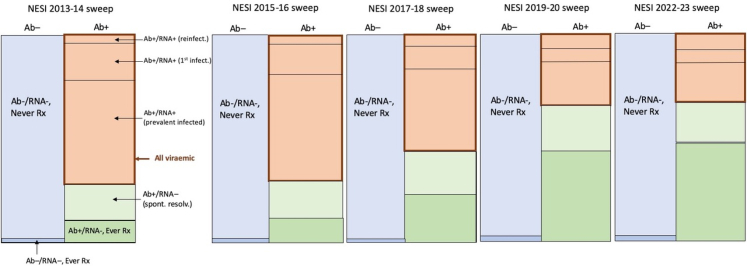
Diagram of changes in the NESI PWID population over four sweeps, according to Ab, RNA, and ever-treated (‘Ever Rx’) status. Acute infections (Ab-/RNA+, never treated) are not shown as they represent a very small proportion of all surveyed PWID. The area enclosed by the thick red border refers to all chronically infected (Ab+/RNA+) PWID. Note: diagram only approximates the actual distributions of Ab, RNA and ever-treated status in the NESI data.

We defined a Markov model to capture basic aspects of the NESI population dynamics, where HCV antibody test result, HCV RNA test result, and self-reported ‘ever treated’ status were used to partition the population into subgroups (see Fig. 1). Membership of population subgroups evolve over time, due to effective DAA treatment, the occurrence of reinfection, and new (primary) infection. The Markov model describes the flow of the NESI population between subgroups over time. The time step was defined as two years (the inter-sweep interval). Although the number of participants varied between sweeps, the modelling approach (like dynamic transmission models) only requires keeping track of the proportions of each subgroup within a sweep. To approximate the degree of uncertainty implicit in the amount of NESI data collected per sweep, the observed proportions are multiplied by a constant number of participants per sweep (*n* = 2200, roughly the average number of NESI participants over the 2013-14, 2015-16, 2017-18, 2019-20, and 2022-23 sweeps). We note that because of this standardisation of participant numbers, the amount of uncertainty will be slightly underestimated for sweeps with fewer than 2200 participants.

In each NESI sweep, the proportion of viraemic (Ab+/RNA+) participants is known (see Supplementary Materials, Table S1); however, this comprises a mixture of proportions from three key subgroups, which are not known: (i) reinfections (i.e., people who had previously resolved their chronic infection, due to successful treatment or spontaneous resolution), (ii) new (chronic) infection (i.e., people who previously had Ab-/RNA-status and had never been treated), (iii) prevalent chronic infection (people who were also chronically infected one sweep earlier; see Fig. 1). The task of the model is to infer these three proportions, given partial knowledge on each of these proportions, for each sweep. From these inferred proportions (i-iii), the incidence rate of new (primary) infection and reinfection (both separately and combined (‘total infection’)) between sweeps can be calculated, with accompanying uncertainty. Inference is carried out in a Bayesian framework to propagate uncertainty associated with the data and prior assumptions to the posterior estimates.

The model is described by the following equations. In each equation, the index *i* refers to NESI sweep (e.g., *i* = 1 for 2013-14). First, the observed total number of viraemic participants per sweep, *n*_*Vir,i*_, is defined as a summation of the (unknown) numbers in each of the three viraemic subgroups (i.e., *n*_*reinf,*i_ + *n*_*1stinf,i*_ + *n*_*previnf,i*_) using the *sum* sampler in JAGS:^19^

(Eq. 1) $$n_{Vir,i}=n_{reinf,i}+n_{1stinf,i}+n_{previnf,i}$$

The number of HCV reinfections in sweep *i* is defined as binomially distributed, with the ‘number of trials’ parameter set to the number of persons who had resolved their infection through antiviral therapy (i.e., either Ab+/RNA-status or Ab-/RNA-status for those who also reported ever being treated for HCV; Ab-/RNA-is included because antibody response can wane following treatment, for a small proportion of individuals^20^^,^^21^; the proportions of PWID who reported ever being treated with a Ab-/RNA-test result in the NESI data are low: range of 0.004 [*n* = 9] to 0.029 [*n* = 67]) in sweep *i–1* (*n*_resolved,i–1_)), and with proportion parameter (*p*_reinf,i_) set to the estimated per-sweep reinfection proportion (Eq. (2a)). A prior on the proportion (or, risk) of HCV reinfection (*p*_reinf,i_) is specified as follows. First, from NESI data, the 2-year (or 3-year, between 2019-20 and 2022-23 only) risk of reinfection was calculated for each sweep as the number of viraemic participants (i.e., testing Ab+/RNA+) among all participants who had reported (i) ever having been treated for HCV, and (ii) that they had cleared their HCV infection after treatment. This we calculated per sweep at: 2013-14: 0.069 (5/73); 2015-16: 0.168 (20/119); 2017-18: 0.157 (39/248); 2019-20: 0.077 (29/378); 2022-23: 0.127 (66/521). (Note that these values represent lower bounds of the risk of reinfection since the previous sweep, as a reinfected person could potentially have been successfully re-treated between survey sweeps). Parameters of a Beta distribution were then fitted to the risk of reinfection for the final four sweeps (2015-16, 2017-18, 2019-20, and 2022-23) only; thus *i* in Eq. (2a) and Eq. (2b) ranges from 2 to 4.

(Eq. 2a) $$n_{reinf,i}\;∼\;Binomial\left(n_{resolved,i-1}{,p}_{reinf,i}\right)$$

(Eq. 2b) $$n_{resolved,i}=\left(n_{Ab+RNA-,everRx,i}+n_{Ab-RNA-,everRx,i}\right)$$

We estimate the 2- (or 3-) year risk of new (primary) HCV infection based on the estimated number of new infections occurring between sweep *i*-1 and sweep *i*. This is estimated for the final four sweeps (2015-16, 2017-18, 2019-20, and 2022-23) only; thus *i* in Eqs. (Eq. 3a), (Eq. 3c) ranges from 2 to 5. The number of primary HCV infections in sweep *i* is defined as binomially distributed (Eq. 3a), with the ‘number of trials’ parameter set to the number of persons who had previously never been infected (i.e., Ab-/RNA-status, with the condition that they did not report ever having been treated, because loss of antibody can also occur following treatment) in sweep *i–1* (*n*_*naive,i–1*_), and with proportion parameter (*p*_1stinf,i_), a model parameter to be inferred, assigned an informative Beta prior based on estimated acute infection incidence rates (with 95% CI) as previously reported using the window period method.^15^

(Eq. 3a) $$n_{1stinf,i}\;∼\;Binomial\left(n_{naive,i-1}{,p}_{1stinf,i}\right)$$

(Eq. 3b) $$n_{naive,i}=n_{Ab-RNA-,neverRx,i}$$

(Eq. 3c) $$p_{1stinf,i}\;∼\;Beta\left(a_{i},b_{i}\right)$$

The number of prevalent HCV infections in sweep *i* (*n*_*previnf,i*_) is defined as the sum of (i) the number of prevalent (=existing) viraemic HCV infections in sweep *i–1* and (ii) the number of incident (=’new’; including both primary infections and reinfections) HCV infections arising in sweep *i–1* (Eq. (4a)); this sum (*n*_*sum*,i–1_) is then adjusted for those clearing their infection (either primary infection or reinfection) through treatment (Eq. (5)). We assume that this parameter is binomially distributed, with the ‘number of trials’ parameter set to the number of viraemic persons in sweep *i–1* (*n*_*previnf*,i–1_), and the proportion parameter set to the proportion estimated to remain viraemic (*prop*_*remainV*ir,i_), which is a function of the proportion undergoing DAA treatment since the previous sweep (Eq. (4b); also see below) and the sustained virologic response (SVR) rate (estimated by Palmateer et al.^7^ using data from Scotland's HCV Clinical database in 2017–18 on people with an SVR test result): 98.2% (95% CI: 97.6–98.7%) (Eq. (4c)). In sensitivity analysis 2, we tested an alternative, lower value, namely, by calculating the midpoint SVR rate between this per-protocol rate and the intention-to-treat (ITT) rate from the same publication: 85.1% (95% CI: 84.2–86.7%).

(Eq. 4a) $$n_{sum,i-1}=\left({n_{previnf,i-1}+n}_{reinf,i-1}+n_{1stinf,i-1}\right)$$

(Eq. 4b) $${prop}_{remainVir,i}=1-\left({prop}_{Rxsince,i}\times prob.SVR\right)$$

(Eq. 4c) prob.SVR∼Beta(139.9,277.2)

(Eq. 5) $$n_{previnf,i}\;∼\;Binomial\left(n_{sum,i-1},{prop}_{remainVir,i}\right)$$

Data on the per-sweep numbers of respondents who reported undergoing DAA treatment for their infection in the past year are used to inform the *propRx*_since,i_ parameters, which are defined as the estimated proportion of viraemic participants reporting any DAA or ‘both’ (i.e., both interferon-based and DAA; a small percentage only) between sweep *i–1* and sweep *i*. (The number of respondents reporting treatment in the past year serves as a proxy for the number treated since the previous sweep.) This value (Supplementary Materials, Table S2) was then divided by the proportion of viraemic persons in the first sweep of each transition (e.g., 0.0805/0.372 = 0.216 and 0.1018/0.302 = 0.337 for the 2015-16 to 2017-18 and the 2017-18 to 2019-20 transitions, respectively; Supplementary Materials, Table S2) to estimate the proportion of viraemic participants in the previous sweep who were treated with DAAs. For the transition between the 2013-14 and 2015-16 sweeps, the calculation was necessarily carried out using data on reporting of pre-DAA era treatment.

The per-sweep viraemic prevalence is defined as the estimated total number of viraemic persons divided by the total number of respondents in sweep *i*:

(Eq. 6) $$n_{Vir,i}=n_{reinf,i}+n_{1stinf,i}+n_{previnf,i}$$

(Eq. 7) $${prev}_{,i}=n_{Vir,i}/N_{i}$$

The proportions of primary infection and prevalent infection for a particular sweep are therefore inferred from the data for the current and previous sweep and the estimated parameters for the previous sweep. For the first sweep only, all components of *n*_*Vir,i*_ (i.e., *n*_1stinf,1_, *n*_reinf,1_, and *n*_previnf,1_) are unknown and must be assigned informative priors. These are (in part) based on published data from the 2011-2012 sweep.^7^ That is, *n*_reinf,1_, *n*_1stinf,1_, and *n*_previnf,1_ are introduced in the model as Poisson distributed variables, with rate parameters (λ) defined as follows:

(Eq. 8a) $$n_{reinf,1}\;∼\;Poisson\left(\lambda_{reinf,1}\right)$$

(Eq. 8b) $$\lambda_{reinf,1}=n_{resolved,0}\times p_{reinf,1}$$

In Eq. (8b), *n*_resolved,0_ refers to the number of participants who had resolved their infection after treatment in sweep ‘0’ (2011-12), as estimated from Palmateer et al.,^7^ and *p*_reinf,1_ is the estimated risk of reinfection occurring between sweeps 0 and 1.

(Eq. 9a) $$n_{1stinf,1}\;∼\;Poisson\left(\lambda_{1stinf,1}\right)$$

(Eq. 9b) $$\lambda_{1stinf,1}=n_{naive,0}\times 0.181$$

In Eq. (9b), *n*_naive,0_ refers to the number of participants in sweep ‘0’ (2011-12) with Ab-/RNA-status who had never been treated, as estimated from Palmateer et al.,^7^ and 0.181 is the estimated risk of primary infection in sweep 2013-14, derived from the incidence rate calculated using the window period method (10 per 100 person-years; 95% CI: 5-15)^15^; see Supplementary Materials for details. The importance of this chosen parameter value we explored in sensitivity analysis 1.

(Eq. 10a) $$n_{previnf,1}\;∼\;Poisson\left(\lambda_{previnf,1}\right)$$

(Eq. 10b) $$\lambda_{previnf,1}=n_{Vir,1}\;-\;\left(n_{reinf,1}+n_{1stinf,1}\right)$$

In Eq. (10b), the mean number of prevalent infections in the 2013-14 sweep, λ_previnf,1_, is defined as the estimated number of viraemic persons (from Palmateer et al.^7^) after subtracting the estimated mean number of reinfections and primary infections (both leading to chronic infection) (Eqs. (Eq. 8b), (Eq. 9b))), in the same sweep.

Finally, a set of functional parameters were defined. First, the estimated 2- (or 3-) year risks of primary infection and reinfection were converted to average incidence rates using Eqs. (Eq. 11a), (Eq. 11b)), with *t* = 2 (*t* = 3 for 2022-23):

(Eq. 11a) $$avg.{incrate}_{1stinf,i}=-log\left(1-p_{1stinf,i}\right)/t$$

(Eq. 11b) $${avg.incrate}_{reinf,i}=-\log\left(1-p_{reinf,i}\right)/t$$

Second, the time at risk (*y*_i_) was calculated from the average incidence rates and the estimated numbers of primary infections and reinfections through simple re-arrangement:

(Eq. 12a) $$y_{1stinf,i}=\frac{n_{1stinf,i}}{{avg.incrate}_{1stinf,i}}$$

(Eq. 12b) $$y_{reinf,i}=\frac{n_{reinf,i}}{{avg.incrate}_{reinf,i}}$$

Next, primary infection and reinfection incidence rates per 100 person-years were calculated as the number of infections divided by time at risk (Eq. (Eq. 13a), (Eq. 13b)):

(Eq. 13a) $${incrate}_{1stinf,i}=\frac{n_{1stinf,i}\;}{y_{1stinf,i}}\times 100$$

(Eq. 13b) $${incrate}_{reinf,i}=\frac{n_{reinf,i}\;}{y_{reinf,i}}\times 100$$

Then, the total infection incidence rate was estimated as the sum of the estimated numbers of primary infection and reinfection, divided by the total time at risk (Eq. (13c)), and then multiplied by 100 to yield the incidence rate expressed per 100 person-years:

(Eq. 13c) $${incrate}_{totalinf,i}=\frac{\left(n_{1stinf,i}{\;+\;n}_{reinf,i}\right)}{\left(y_{1stinf,i}{\;+\;y}_{reinf,i}\right)}\times 100$$

Finally, we estimate the posterior difference in the incidence rates of primary infection, reinfection, and total infection between sweeps 2 and 3, between sweeps 2 and 4, and between sweeps 2 and 5 (Eq. (Eq. 14a), (Eq. 14b), (Eq. 14c)); in these equations the index j varies from 3 to 5.

(Eq. 14a) $${diff}_{1stinf,j}=\left({incrate}_{1stinf,2}{-incrate}_{1stinf,j}\right)$$

(Eq. 14b) $${diff}_{reinf,j}=\left({incrate}_{reinf,2}{-incrate}_{reinf,j}\right)$$

(Eq. 14c) $${diff}_{totalinf,j}=\left({incrate}_{totalinf,2}{-incrate}_{totalinf,j}\right)$$

### Implementation

Estimation of the posterior distributions was conducted using Markov-chain Monte-Carlo methods. After discarding the first 6000 iterations as burn-in, 3000 samples from the posterior distribution of each chain were taken to allow inference of the model parameters. Three chains were run, and results examined for adequate mixing and convergence. We report posterior median estimates and define 95% credible intervals (CrIs) using the 2.5% and 97.5% quantiles of the posterior distribution. Sampling was carried out using JAGS version 4.3.0,^19^ accessed via the runjags package in R version 4.2.3.^22^

### Role of the funding source

Funding sources had no involvement in study design, the collection, analysis, and interpretation of data, the writing of the report, or in the decision to submit for publication.

## Results

### Characteristics of the study population

The study population consisted of an average of 2138 individuals per NESI sweep during the period 2015 to 2023 (Table 1). The average age of NESI participants gradually increased over this time period (median of 37.7 years rising to 43.5 years), but the proportion male was relatively stable (70%–69%). Across survey years, the age and sex distributions of NESI respondents were broadly comparable to those estimated for the entire population of people dependent on opioids in Scotland (Supplementary Materials, Table S5). The proportion of respondents prescribed methadone within the last 6 months varied across surveys (68–81%) (Table 1). Considering participants who had visited the recruitment site for injecting equipment (i.e., excluding those who had attended specifically for OAT), the proportion prescribed methadone was lower (63–69%) across surveys and consistent with the proportion on OAT in the last year for the estimated entire population dependent on opioids (Supplementary Materials, Table S5). Reported recent injecting (within the last 6 months) decreased, from 82% in 2015-16 to 61% in 2022-23. The reported receipt of HCV therapy in the past year generally increased over time, from 2·9% in 2015-16 to 10% in 2019-20 and 9% in 2022-23. Chronic HCV infection prevalence rapidly decreased, from 37% in 2015-16 to 15% in 2022-23 (Table 1).

**Table 1:** Characteristics of PWID surveyed across Scotland as part of NESI, sweeps 2015–16 through 2022–23.

	NESI sweep			
	2015-16	2017-18	2019-20	2022-23
Respondents included; *n*	2439	1876	2311	1926
Age (years); *median*	37.7	40.2	40.4	43.5
Male sex; *prop.*	0.704	0.727	0.717	0.685
Injected in last 6 months; *prop.*	0.815	0.681	0.673	0.613
Received methadone in last 6 months; *prop.*	0.775	0.808	0.799	0.675
Received methadone in last 6 months, among those reporting injecting in last 6 months; *prop.*	0.765	0.791	0.787	0.684
Ever treated for HCV; *n* (*prop.).*	203 (0.083)	352 (0.188)	489 (0.212)	677 (0.352)
Treated for HCV in last year; *n* (*prop.)*	70 (0.029)	151 (0.081)	235 (0.102)	176 (0.091)
Chronic HCV infection (Ab+/RNA+); *n (prop.)*	908 (0.372)	567 (0.302)	444 (0.192)	286 (0.148)
Cleared HCV infection (Ab+/RNA–); *n (prop.)*	431 (0.177)	464 (0.247)	819 (0.354)	868 (0.451)
Never HCV infected (Ab-/RNA-); *n (prop).*	1068 (0.438)	794 (0.423)	981 (0.424)	734 (0.381)

### Inferred HCV incidence rates

**Fig. 2:**
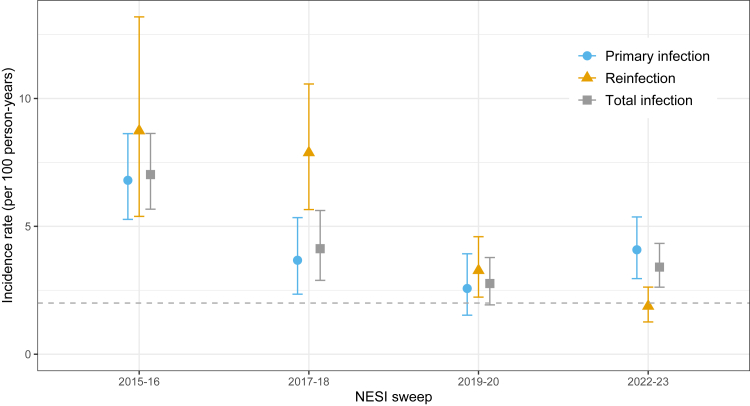
Model-estimated incidence rates of primary infection, reinfection and total HCV infection in the four sweeps 2015-16, 2017-18, 2019-20, and 2022-23. The WHO target is shown as a horizontal dashed line.

The incidence rates of total new infections (per 100 person-years), summing primary HCV infection and reinfection, for the 2015-16, 2017-18, 2019-20, and 2022-23 sweeps were estimated at 7.0 (95% credible interval (CrI): 5.7–8.6), 4.1 (95% CrI: 2.9–5.6), 2.8 (1.9–3.8), and 3.4 (2.6–4.3) HCV infections per 100 person-years, respectively (Fig. 2). Reinfection incidence rates were estimated at 8.7 (95% CrI: 5.4–13.2), 7.9 (5.7–10.6), 3.3 (2.2–4.6), and 1.9 (1.3–2.6) per 100 person-years, for the 2015-16, 2017-18, 2019-20, and 2022-23 sweeps. The incidence rates of primary HCV infection for the 2015-16, 2017-18, 2019-20, and 2022-23 sweeps were estimated at 6.8 (95% CrI: 5.3–8.6), 3.7 (2.3–5.3), 2.6 (1.5–3.9), and 4.1 (3.0–5.4) per 100 person-years, respectively.

### Estimated changes in HCV incidence rates over time

Compared with 2015-16, model-estimated total, re-infection and primary infection incidence rates decreased in 2022-23 by 51% (absolute decrease of 3.6 per 100 person-years; 95% CrI: 2.0–5.3), 78% (decrease of 6.9; 95% CrI: 3.4–11.3), and 40% (decrease of 2.7; 95% CrI: 0.7–4.8), respectively. Model-estimated incidence rates had also significantly decreased between 2015–16 and 2019–20: total infection by 61% (absolute decrease of 4.2 per 100 person-years; 95% CrI: 2.6–6.1), reinfection by 63% (decrease of 5.4; 95% CrI: 1.8–10.0), and primary infection by 62% (decrease of 4.2: 95% CrI: 2.2–6.4). There were statistically significant reductions in the incidence rates of total and primary infection only between the reference sweep 2015-16 and the 2017-18 sweep: 2.9 (95% CrI: 0.8–4.9) and 3.1 (95% CrI: 0.9–5.4) infections per 100 person-years, respectively. Computed Bayesian *p*-values indicated that the model-estimated total infection incidence rate was below 2% with a mean probability of 0.037 for the 2019-20 sweep only; for 2017-18 and 2022-23 this mean probability was 0.0.

### Sensitivity analyses

As the incidence rate of primary HCV infection in sweep 2013-14 is not inferred, but must be provided to the model as data, the inferred incidence rates in subsequent sweeps may be sensitive to the choice of this initial value. Results of sensitivity analysis 1 using a lower (5/100 person-years) and higher value (15/100 person-years) than the chosen initial value (10/100 person-years) revealed that there was no influence on the primary or total infection incidence rates inferred for later sweeps (Supplementary Materials, Table S3).

Results of sensitivity analysis 2 using a lower SVR rate (the midpoint of the baseline (per-protocol) and ITT rates) had a relatively small influence on the reinfection and total infection incidence rates; for instance, the total infection incidence rates inferred for the 2019-20 and 2022-23 sweeps decreased to 2.3 and 2.8 per 100 person-years, respectively (Supplementary Materials, Table S4).

### Relation to population of people dependent on opioids

We assumed that NESI respondents represent PWID attending services providing needle syringe programmes (NSP), as recruitment is at sites providing injecting equipment and targets those actively injecting. While the derived HCV incidence estimates likely apply to individuals accessing NSP, they may not generalise to the whole population of people dependent on opioids. The latter involves individuals who inject drugs but who do not access NSP and related harm reduction services; however, this is likely to be a small minority in Scotland where NSP is freely and widely available. Moreover, in Scotland, this includes a substantial number of people on long-term OAT who may have ceased injecting,^18^ with consequently minimal risk of HCV primary infection or reinfection (appreciating transmission may still occur through routes other than injecting drug use). A rough estimate of the proportion of the whole population of people dependent on opioids in Scotland that would need to be at zero or low risk of acquiring infection to achieve the WHO target of ≤2 infections per 100 person-years can be made using our model-based total infection incidence rate estimate for 2022-23 (3.4 per 100 person-years). In the NESI 2022-23 population, 39% of all respondents reported not having injected in the past 6 months and are thus considered at low risk of HCV acquisition. Under the strong assumption that NESI respondents who have recently injected are representative of the active injectors in the population of people dependent on opioids, the WHO incidence target would be achieved when an estimated 64% of this population had ceased injecting, shown by solving for *x* in the expression: (1–0.39)/(1–*x*) = 3.4/2.0.

## Discussion

To evaluate progress against the WHO targets regarding the elimination of HCV as a public health issue, our modelling approach estimated primary, reinfection, and total infection incidence rates among PWID surveyed across Scotland over four NESI sweeps, and indicated declining HCV incidence rates over time corresponding to declines in HCV chronic prevalence. Compared with the reference 2015-16 sweep, the model-estimated total infection incidence rate significantly decreased in 2019-20 and in 2022-23 (to 2.8 and 3.4 per 100 person-years, respectively, a decrease of 51–61%), and the incidence of primary infection significantly decreased between 2015-16 and 2017-18, and then further to a low of 2.6 per 100 person-years in 2019-20. These model-estimated incidence rates, while not yet meeting the WHO HCV elimination target of a total infection incidence of ≤2 per 100 person-years among PWID for Scotland as a whole, represent important progress – incidence rates of 2–3 per 100 person-years are historically low – and suggest that the target is achievable in the near future.^11^

The significant decrease in all three HCV incidence rate measures between 2015-16 and 2022-23 is consistent with a constant reduction in HCV transmission. Considering the 2022-23 data more closely (Supplementary Materials, Table S1), there are two notable observations: (i) the proportion of respondents susceptible to primary infection (i.e., Ab–/RNA-, never treated) dropped to 0.38, whereas this proportion had been relatively stable across the preceding four sweeps at around 0.42–0.43; and (ii) the proportion of chronically infected (i.e., Ab+/RNA+) decreased to a low of 0·148. Taken together, these results are compatible with both a decrease in new initiates to injecting (i.e., relatively fewer susceptible persons participating in the 2022-23 sweep), and the positive impact of scaling up TasP on transmission rates (as demonstrated by reductions in the prevalence of chronic HCV infection associated with TasP scale-up^7^^,^^8^). Finally, Fig. 2 suggests a potential rise in the incidence of primary and total infection between 2019-20 and 2022-23; although the overlapping 95% CrIs do not support a genuine increase, this observation warrants continued monitoring and effort to prevent transmission, particularly in context of changing temporal trends in drugs injected – involving a marked rise in reported injection of powder cocaine among NESI participants from 37% in 2019-20 to 60% in 2022-23 – in Scotland.^12^

Model-estimated incidence rates of reinfection were observed to be consistently higher than those for primary infection across the first three sweeps, 2015-16 through 2019-20 (Fig. 2), but because the percentages of respondents with treatment-resolved infection were calculated (from NESI data) at 5.3%, 14.4%, and 18.5% across the respective sweeps, model-estimated reinfection rates translated to fewer than 20 reinfected persons per sweep.

Strengths of this study include the relatively large coverage of the NESI sweeps: the data are extensive enough to permit estimation of most model parameters with reasonable confidence, with some exceptions (such as the reinfection rate, which is derived from relatively small numbers (Eq. (8b))). Several important assumptions and limitations underlie the modelling approach. First, each of the four sweeps was assumed to be equally representative of the underlying PWID population accessing services providing injecting equipment (including in respect of demographic and injecting risk profile). The changing demographics (i.e. increasing age) of the NESI study population over successive surveys was consistent with that of the estimated total population dependent on opioids,^18^ providing support for this assumption. Second, the population was treated as stable in overall size (i.e., between successive sweeps, people who ceased injecting or who had died were ‘replaced’ by the same number of new initiates), which was also consistent with population size estimates for people dependent on opioids in Scotland for the period 2015-2023.^18^ Third, and as a consequence of the previous assumption, the NESI sample size is modelled as constant between sweeps; this does not imply that the demographic profile remains constant. The modelled incidence rates over time thus capture overall changes for the study population, stemming from the impact of DAA treatment on reduced prevalence of infection and from changes in demographic or injecting risk profile. Fourth, there is likely some degree of mis-classification in self-reported ‘ever treated’ status, potentially due to recall bias. Fifth, due to the dependencies between model parameters and data in successive sweeps (see Eq. (Eq. 2a), (Eq. 2b), (Eq. 3a), (Eq. 3b), (Eq. 3c), (Eq. 4a), (Eq. 4b), (Eq. 4c), (Eq. 5), (Eq. 6), (Eq. 7), (Eq. 8a), (Eq. 8b), (Eq. 9a), (Eq. 9b)), incidence rate estimates in later sweeps may be dependent on assumptions made for the first sweep. Namely, the initial value for the primary infection risk in 2013-14 was derived from the acute HCV infection incidence rate for the same sweep, which had been calculated from data using the window method.^15^ However, sensitivity analysis results indicated that the estimated primary infection incidence rates for 2015-16, 2017-18, 2019-20, and 2022-23 were not influenced by this initial value; decreasing or increasing the assumed primary infection incidence rate from 2013 to 14 by ±5/100 person-years did not appreciably change the estimates for later sweeps.

Sixth, though we assume that the NESI population represents individuals attending services providing NSP and are thus likely a higher risk population of PWID, our incidence estimates may therefore not generalise to the whole population of people dependent on opioids; we currently lack evidence to re-weight these estimates. In Scotland, almost 2/3 of the opioid dependent population are on OAT during the year,^18^ but evidence on the proportion not injecting (i.e., thus at low risk of acquiring HCV) is missing. Seventh, we specified a per-protocol SVR rate (98.2%), as this was the most conservative option; sensitivity analysis indicated that substitution of a lower alternative rate (85.1%) did not appreciably affect our results or conclusions. Finally, we necessarily assumed that the number of respondents reporting HCV therapy in the past year serves as a suitable proxy for the proportion of respondents receiving HCV therapy since the previous sweep. Given the two-year gap between four of the sweeps, and the three-year gap between 2019-20 and 2022-23, the effect of a too-low value for the proportion treated since the previous sweep would be to underestimate the true proportion receiving HCV therapy and thus to overestimate the model parameters for the per-sweep proportions who remain viraemic (cf. Eq. (4b)), with consequent error in the estimated reinfection incidence rates (i.e., biased to a lower value).

Previous work in England and Scotland has shown that intensity of HCV treatment scale-up and subsequent impact on chronic HCV and estimated incidence can vary regionally, with some sites expected to achieve WHO elimination targets earlier than others, including Tayside and Bristol.^8^^,^^10^^,^^11^ There are few national-level estimates, however, of HCV incidence considering both primary infection and re-infection. Global reviews have suggested only small reductions in the incidence of primary HCV infection among PWID, with a pooled estimate of (primary) infection incidence of 12.1 per 100 person-years, based on studies published in the period 2000-2022.^23^ Reliance on serological markers (i.e., the window period method applied to the number of cases testing HCV Ab-positive and RNA-positive) lacks robustness; due to low numbers of events uncertainty in these estimates is very high,^15^ and reinfection cannot be captured.

The decrease in Scotland-wide HCV incidence rates from 2015-16 to 2019-20 inferred using the present method is consistent with modelled (with respect to a counterfactual) and observed declines in HCV prevalence among PWID over a similar time-frame in England and Scotland.^8^^,^^10^ This is not surprising, given the theoretical basis for a decrease in the force of infection (transmission intensity) in closed populations when infectious persons are ‘returned’ to the susceptible compartment through effective treatment, a characteristic of NESI populations with active TasP initiatives and relatively low rates of new PWID entering the population. However, there are problems with the use of force of infection models applied to HCV antibody by age-group, as the incidence estimates generated by these models are not contemporary,^24^ and therefore it may take longer to detect declines in HCV incidence. Thus, the model presented here may offer an easier route for countries/regions to generate HCV incidence estimates from similar cross-sectional bio-behavioural survey data. Alternative approaches to estimating incidence using dynamic mathematical modelling can be complex and highly intensive (in terms of the required computing power and development time) to arrive at a fully calibrated model. In future, we plan to compare this method with a national-level dynamic mathematical model for Scotland.

In summary, using an innovative modelling approach, this study has inferred HCV incidence rates from repeated cross-sectional survey data in Scotland, and demonstrated significant reductions, on the order of 40–78%, in the incidence of primary infection, reinfection, and total HCV infection among PWID between 2015-16 and 2022-23. While the WHO absolute HCV incidence target of ≤2 new infections per 100 person-years may not yet have been met across the country, the estimated reduction in transmission following major scale-up of DAAs in this high-risk PWID population provides compelling evidence of TasP. Sustained effort is required – capitalising on the progress to date and optimising testing and harm reduction coverage in community settings such as drug services and prisons – to achieve and maintain HCV elimination among PWID.

## Contributors

SAM and SJH designed the study. SAM, NP, SJH, and MH wrote the first version of the manuscript, with critical review provided by AM, RNG, STB, and JFD. SAM was responsible for conducting the model-based analysis. All authors contributed to the interpretation of results and discussion of the context, and approved submission of the final version. SM and NP have directly accessed and verified the data reported in the manuscript. All authors confirm that they had full access to all the data in the study and accept responsibility to submit for publication.

## Data sharing statement

Data used in the modelling analysis presented in this manuscript are either provided in the accompanying tables/text or in the cited publicly-available reports.

## Declaration of interests

Regarding support for the manuscript, SAM, SJH, AM, MH and NP report institutional research grant funding from NIHR and Public Health Scotland. MH has received support to attend a Viral Hepatitis Prevention Board meeting and is a Trustee for the Society of the Study of Addiction. SB has received payments from Gilead for presentations and Advisory Board participation. JD reports institutional grant funding from Gilead, Abbvie and Intelligent Ultrasound; consulting fees from Intelligent Ultrasound and Novo Nordisk; payment for presentations from Abbvie and Gilead; membership of Trial Data Monitoring Committee for NIHR; Trustee for the British Society of Gastroenterology; and copyright (but no financial return or patent) for Inventor of Intelligent LFT.
