# Supplementary material for: Estimating incidence rates of primary infection and reinfection with hepatitis C virus among people who inject drugs in Scotland: a model-based analysis of repeated cross-sectional survey data
Source: Lancet Reg Health Eur. 2025 Oct 28;60:101505. doi: 10.1016/j.lanepe.2025.101505 (PMC12605865; doi:10.1016/j.lanepe.2025.101505)
Supplement: Multimedia component 1 [file mmc1.docx]

**SUPPLEMENTARY MATERIALS**

Estimating the incidence rates of primary infection and reinfection with hepatitis C virus from repeated cross-sectional bio-behavioural survey data: a statistical model-based approach

*Scott A McDonald, Norah Palmateer, Andrew McAuley, Rory N Gunson, Stephen T Barclay, John F Dillon, Matthew Hickman, Sharon J Hutchinson*

**Calculation of acute infection incidence using the window period method**

The 2-year risks of primary infection was calculated from the estimated incidence rate derived using the window period method (PHS, 2022) as follows:

- The window period method combines data on acute infection (number of persons testing Ab-/RNA+) and an estimate of the time window between transmission and the detection of HCV antibody.
- As an example, for the 2015-16 sweep this was 11.4 per 100 person-years, or 0.114 per person-year.
- The 2-year risk of primary infection in 2015-16 is then calculated as: [1-exp(-0.114*2)] = 0.204

**Table S1. Partitioning of the NESI PWID population in each of five sweeps according HCV Ab and RNA status.**

|  | *2013-14* | | *2015-16* | | *2017-18* | | *2019-20* | | *2022-23* | |
| --- | --- | --- | --- | --- | --- | --- | --- | --- | --- | --- |
|  | **Ab-neg** | **Ab-pos** | **Ab-neg** | **Ab-pos** | **Ab-neg** | **Ab-pos** | **Ab-neg** | **Ab-pos** | **Ab-neg** | **Ab-pos** |
| RNA-pos | – | 0.341 | – | 0.372 | – | 0.302 | – | 0.192 | – | 0.148 |
| RNA-neg,  *never   treated* | 0.434 | *n.c.* | 0.438 | *n.c.* | 0.423 | *n.c.* | 0.424 | *n.c.* | 0.381 | *n.c.* |
| RNA-neg,  *ever   treated* | 0.004 | *n.c.* | 0.013 | *n.c.* | 0.027 | *n.c.* | 0.029 | *n.c.* | 0.020 | *n.c.* |
| RNA-neg,  *all* | *n.c.* | 0.221 | *n.c.* | 0.177 | *n.c.* | 0.247 | *n.c.* | 0.354 | *n.c.* | 0.451 |

*Note*. ‘–‘ indicates combination not present in data; *n.c.* = not calculated separately

**Table S2. Details of the calculation of *propRx*_since,i_ parameter values from NESI data.**

|  | *2013-14* | *2015-16* | *2017-18* | *2019-20* | *2022-23* |
| --- | --- | --- | --- | --- | --- |
| Viraemic participants; *n* (prop.) | 749 (0.341) | 908 (0.372) | 567 (0.302) | 444 (0.192) | 286 (0.148) |
| Participants who reported being treated with DAAs in last year; [*n/N*] (prop.) | –– | [70/2439] (0.0287) ^[1]^ | [151/1876] (0.0805) | (0.1018) ^[2]^ | [176/1926] (0.0914) |
| Inferred prop. of viraemic participants treated with DAAs since previous sweep ^[3]^ | –– | 0.084 *(=0.0287/0.341)* | 0.216 *(=0.0805/0.372)* | 0.337 *(=0.1018/0.302)* | 0.476 *(=0.0914/0.192*) |

[1] For 2015-16, this value reflects a mix of interferon-containing and interferon-free (DAAs) therapies, because the first DAA (sofosbuvir) was available following licensing for use in Scotland from approximately November 2014.

[2] For 2019-20, n and N from the NESI 2022 report (Table 1.4) (PHS, 2022) were used to calculate the proportion treated in the last year (i.e., 0.1018; 248/2435).

[3] This estimate is labelled ‘inferred since previous sweep’ because it is based on the NESI question regarding HCV treatment in last year. In calculating this proportion, it is assumed that only viraemic participants were treated.

**Table S3. Results of sensitivity analysis 1: the effect of varying the prior on the primary infection incidence rate in 2013-14 on the inferred primary and total infection incidence (in new infections per 100 person-years) in later sweeps**

|  | Inferred primary infection incidence rate, per 100 person-years (95% CI) | | | |
| --- | --- | --- | --- | --- |
| Prior on primary infection incidence rate, 2013-14 | **2015-16** | **2017-18** | **2019-20** | **2022-23** |
| Baseline value (10/100 person-years) | 6.8 (5.3-8.6) | 3.7 (2.3-5.3) | 2.6 (1.5-3.9) | 4.1 (3.0-5.4) |
| Lower value (5/100 person-years) | 6.8 (5.2-8.6) | 3.7 (2.3-5.4) | 2.5 (1.5-3.9) | 4.1 (3.0-5.3) |
| Higher value (15/100 person-years) | 6.8 (5.2-8.6) | 3.7 (2.4-5.3) | 2.5 (1.5-4.0) | 4.1 (3.0-5.4) |
|  | **Inferred total infection incidence rate, per 100 person-years (95% CI)** | | | |
| Prior on primary infection incidence rate, 2013-14 | **2015-16** | **2017-18** | **2019-20** | **2022-23** |
| Baseline value (10/100 person-years) | 7.0 (5.7-8.6) | 4.1 (2.9-5.6) | 2.8 (1.9-3.8) | 3.4 (2.6-4.3) |
| Lower value (5/100 person-years) | 7.0 (5.6-8.6) | 4.1 (2.9-5.7) | 2.7 (1.9-3.8) | 3.4 (2.6-4.3) |
| Higher value (15/100 person-years) | 7.0 (5.6-8.6) | 4.1 (2.9-5.6) | 2.7 (1.9-3.8) | 3.4 (2.6-4.3) |

**Table S4. Results of sensitivity analysis 2: comparing the baseline SVR % against an alternative (midpoint of per-protocol and ITT estimates), for the inferred reinfection and total infection incidence rates (in new infections per 100 person-years)**

|  | Inferred reinfection incidence rate, per 100 person-years (95% CI) | | | |
| --- | --- | --- | --- | --- |
| SVR % | **2015-16** | **2017-18** | **2019-20** | **2022-23** |
| Per-protocol value (98.2%)^[1]^ | 8.7 (5.4-13.2) | 7.9 (5.7-10.6) | 3.3 (2.2-4.6) | 1.9 (1.3-2.6) |
| Midpoint of per-protocol and ITT values^[1]^ (85.1%) | 8.6 (5.2-13.1) | 7.7 (5.5-10.5) | 3.1 (2.1-4.4) | 1.8 (1.2-2.6) |
|  | **Inferred total infection incidence rate, per 100 person-years (95% CI)** | | | |
| SVR % | **2015-16** | **2017-18** | **2019-20** | **2022-23** |
| Per-protocol value (98.2%)^[1]^ | 7.0 (5.7-8.6) | 4.1 (2.9-5.6) | 2.8 (1.9-3.8) | 3.4 (2.6-4.3) |
| Midpoint of per-protocol and ITT values^[1]^ (85.1%) | 6.6 (5.3-8.1) | 3.5 (2.4-4.9) | 2.3 (1.6-3.2) | 2.8 (2.2-3.7) |

[1] Estimates from Palmateer et al. (2021).

**Table S5. Comparison of characteristics of (a) NESI participants with (b) estimated prevalence of people dependent on opioids in Scotland***

| Year | Total (N) | Male sex (%) | Age 15-34 years (%) | Age 35-49 years (%) | Age 50-64 years (%) | On OAT in last year (%)** |
| --- | --- | --- | --- | --- | --- | --- |
| (a) NESI participants | | | | | | |
| 2015-16 | 2,439 | 70% | 35% | 59% | 6% | 64% |
| 2017-18 | 1,876 | 73% | 22% | 66% | 12% | 69% |
| 2019-20 | 2,311 | 72% | 21% | 64% | 14% | 66% |
| 2022-23 | 1,926 | 69% | 18% | 59% | 22% | 63% |
| (b) Estimated population of people dependent on opioids | | | | | | |
| 2015-16 | 46,500 | 69% | 29% | 59% | 12% | 63% |
| 2017-18 | 44,800 | 69% | 27% | 60% | 13% | 66% |
| 2019-20 | 47,100 | 69% | 25% | 59% | 16% | 61% |
| 2022-23 | 43,400 | 69% | 21% | 56% | 24% | 66% |
|  |  |  |  |  |  |  |

*Source: Public Health Scotland. *Estimated Prevalence of Opioid Dependence in Scotland, 2014/15 to 2022/23*, March 2025. <https://publichealthscotland.scot/media/32049/2025-03-18-opioid-prevalence-report.pdf>

** Relating to those NESI respondents who had attended the recruitment site for injecting equipment (i.e., excluding those who had attended for OAT). For all NESI respondents (i.e., including those who had attended for OAT), the proportions on OAT were 77% in 2015-16, 79% in 2017-18, 79% in 2019-20 and 68% in 2022-23

**References**

Palmateer NE, McAuley A, Dillon JF, et al. Reduction in the population prevalence of hepatitis C virus viraemia among people who inject drugs associated with scale‐up of direct‐acting anti‐viral therapy in community drug services: real‐world data. Addiction 2021;116(10):2893-2907.

Public Health Scotland (PHS), Glasgow Caledonian University and the West of Scotland Specialist Virology Centre. *The Needle Exchange Surveillance Initiative (NESI): Prevalence of blood-borne viruses and injecting risk behaviours among people who inject drugs attending injecting equipment provision services in Scotland, 2008 to 2020*. Glasgow: Public Health Scotland, March 2022.

**JAGS code**

model {

# Priors on 2- (or 3-) year risks

for(i in 2:5) { # loop over sweeps 2-5 only

p.1stinf[i] ~ dbeta(p.1stinf.a[i],p.1stinf.b[i])

p.reinf[i] ~ dbeta(p.reinf.a[i],p.reinf.b[i])

}

p.SVR ~ dbeta(p.SVR.a,p.SVR.b)

# Specify priors on number of infections in 1st sweep (2013/14), as all are unknown

n.previnf[1] ~ dpois(lambda.previnf.1)

n.reinf[1] ~ dpois(lambda.reinf.1)

n.1stinf[1] ~ dpois(lambda.1stinf.1)

# Sum of viraemic (Ab+/RNA+) in sweep i = reinfections + new infections + prevalent infections n.Abpos.RNApos[1] ~ sum(n.reinf[1],n.1stinf[1],n.previnf[1])

n.viraemic[1] <- (n.reinf[1] + n.1stinf[1] + n.previnf[1])

prev[1] <- n.viraemic[1] / N[1]

# Likelihood

for(i in 2:5) { # loop over sweeps 2-5 only

# No. reinfections in sweep i is function of no. resolved in sweep i-1

n.reinf[i] ~ dbin(p.reinf[i],n.resolved[i-1])

# No. primary infections in sweep i is function of no. susceptible in sweep i-1

n.1stinf[i] ~ dbin(p.1stinf[i],n.Abneg.RNAneg.NeverRx[i-1])

# No. prevalent (=existing) infections in sweep i is function of no. viraemic in sweep i-1

n.sum[i-1] <- (n.previnf[i-1] + n.1stinf[i-1] + n.reinf[i-1])

n.previnf[i] ~ dbin(prop.remain.chronic[i], n.sum[i-1])

# Prop. treated since the previous sweep and prop. remaining viraemic

p.Rxsince[i] ~ dbeta(p.Rxsince.a[i],p.Rxsince.b[i])

prop.remain.chronic[i] <- (1-(p.Rxsince[i] * p.SVR))

# Sum of viraemic (Ab+/RNA+) in sweep i = reinfections + new infections + prevalent infections

n.Abpos.RNApos[i] ~ sum(n.reinf[i],n.1stinf[i],n.previnf[i])

# Define prevalence of viraemic (Ab+/RNA-) infection in sweep i

n.viraemic[i] <- (n.reinf[i] + n.1stinf[i] + n.previnf[i])

prev[i] <- n.viraemic[i] / N[i]

# Functional nodes for time at risk and incidence rate (infections per 100 pyrs)

# NB. sweep.interval[i] is defined as number of years since previous sweep

avg.incrate.1stinf[i] <- -log(1-p.1stinf[i])/sweep.interval[i]

y.1stinf[i] <- (n.1stinf[i]/avg.incrate.1stinf[i])

incrate.1stinf[i] <- n.1stinf[i]/y.1stinf[i]*100

avg.incrate.reinf[i] <- -log(1-p.reinf[i])/ sweep.interval[i]

y.reinf[i] <- (n.reinf[i]/avg.incrate.reinf[i])

incrate.reinf[i] <- n.reinf[i]/y.reinf[i]*100

# Functional node for total incidence rate

incrate.totalinf[i] <- (n.1stinf[i] + n.reinf[i]) / (y.1stinf[i] + y.reinf[i])*100

}

for(i in 1:4) { # loop over sweeps 1-4 only

n.resolved[i] <- (n.Abpos.RNAneg.EverRx[i] + n.Abneg.RNAneg.EverRx[i])

}

# Nodes for difference in 1stinf, reinf, and totalinf inc rates between sweeps 2&3, 2&4 and 2&5

for(j in 3:5) {

diff.incrate.1stinf[j] <- incrate.1stinf[2] - incrate.1stinf[j]

diff.incrate.reinf[j] <- incrate.reinf[2] - incrate.reinf[j]

diff.incrate.totalinf[j] <- incrate.totalinf[2] - incrate.totalinf[j]

}

}
